# A predicted protein interactome for rice

**DOI:** 10.1186/1939-8433-5-15

**Published:** 2012-07-02

**Authors:** Chai-Ling Ho, Yingzhou Wu, Hong-bin Shen, Nicholas J Provart, Matt Geisler

**Affiliations:** Faculty of Biotechnology and Biomolecular Sciences, Universiti Putra Malaysia, 43400 UPM-Serdang, Selangor Malaysia; Department of Cell & Systems Biology/Centre for the Analysis of Genome Evolution and Function, University of Toronto, Toronto, ON M5S 3B2 Canada; Department of Automation, Shanghai Jiao Tong University, No. 800 Dongchuan Road, Shanghai, 200240 China; Department of Cell & Systems Biology/Centre for the Analysis of Genome Evolution and Function, University of Toronto, Toronto, ON M5S 3B2 Canada; Department of Plant Biology, Southern Illinois University Carbondale, 1125 Lincoln Ave., Life Science II, Carbondale, IL 62901-6509 USA

## Abstract

**Background:**

Protein-protein interactions (PPIs) create the steps in signaling and regulatory networks central to most fundamental biological processes. It is possible to predict these interactions by making use of experimentally determined orthologous interactions in other species.

**Results:**

In this study, prediction of PPIs in rice was carried out by the interolog method of mapping deduced orthologous genes to protein interactions supported by experimental evidence from reference organisms. We predicted 37112 interactions for 4567 rice proteins, including 1671 predicted self interactions (homo-interactions) and 35441 predicted interactions between different proteins (hetero-interactions). These matched 168 of 675 experimentally-determined interactions in rice. Interacting proteins were significantly more co-expressed than expected by chance, which is typical of experimentally-determined interactomes. The rice interacting proteins were divided topologically into 981 free ends (proteins with single interactions), 499 pipes (proteins with two interactions) and 3087 hubs of different sizes ranging from three to more than 100 interactions.

**Conclusions:**

This predicted rice interactome extends known pathways and improves functional annotation of unknown rice proteins and networks in rice, and is easily explored with software tools presented here.

**Electronic supplementary material:**

The online version of this article (doi:10.1186/1939-8433-5-15) contains supplementary material, which is available to authorized users.

## Background

Protein-protein interactions (PPIs) are essential for many fundamental biological processes. With the advent of high-throughput approaches, genome-wide networks of PPIs have been generated in *Saccharomyces cerevisiae* (Uetz et al., 2000; Miller et al., 2005; Gandhi et al., 2006), *Drosophila melanogaster* (Giot et al., 2003), *Caenorhabditis elegans* (Li et al., 2004), *Homo sapiens* (Rual et al., 2005) and other organisms. Recently, a large scale map of 6200 PPIs was completed for *Arabidopsis* (Arabidopsis interactome mapping consortium, 2011). Another medium-scale yeast two-hybrid screen on proteins involved in the two-component signaling pathway of *Arabdidopsis thaliana* has revealed 160 interactions of which 136 were novel (Dortay et al., 2008). Networks of rice genes associated with stress response, seed development and cell cycle mediated by cyclin were built from the results generated from yeast two hybrids (Cooper et al., [Bibr CR11][Bibr CR12]). In addition, a rice kinase-protein interaction map of 116 representative rice kinases and their interacting proteins was generated from the results of yeast two hybrids (Ding et al., 2009).

Prediction of PPIs is made possible in organisms lacking experimental determination of PPIs using the PPI networks established in reference organisms. In this approach, orthologous genes are deduced using prediction algorithms and mapped to protein interactions supported by experimental evidence from reference organisms retrieved from publicly available databases such as Biomolecular Interaction Network Database (BIND; Bader et al., 2001), Molecular Interaction Database (MINT; Zanzoni et al., 2002; Ceol et al., 2009), Munich Information Center for Protein Sequences (MIPS; Pagel et al., 2005), Database of Interacting Proteins (DIP; Salwinski et al., 2004), IntAct (http://www.ebi.ac.uk/intact; Aranda et al., 2010) and Biological General Repository for Interaction Data sets (BioGRID; Breitkreutz et al., 2008). Using this approach, a predicted interactome of *Arabidopsis thaliana* was made consisting 1159 high confidence, 5913 medium confidence and 12907 low confidence interactions. This was established using a confidence scoring based method on the number of different data sets in which the interaction was recorded, the number of different types of experiments supported the interactions, and the number of species in which the interaction was discovered (Geisler-Lee et al., 2007). In addition, the data on subcellular localization and co-expression of interacting proteins were integrated into the deduction of PPIs to strengthen the confidence of the resulting predicted interactome. The predicted interactome in Arabidopsis revealed that many of the most highly conserved proteins were also the most highly connected hubs involved in important signaling complexes, and revealed the preservation of original functions of nuclear-located pathways in non-photosynthetic reference organisms in the chloroplasts of higher plants post endosymbiosis (Geisler-Lee et al., 2007). The Arabidopsis predicted interactome has enabled researchers to fruitfully generate and test network and protein interaction hypotheses (e.g. Liu and Howell 2010, Gu et al. 2008).

In this study, a similar approach was used to predict the interactome of rice with the aim to expand the current understanding of PPIs in monocot based on our predicted interactome. A second goal is to provide a tool that leads to useful hypothesis generation.

## Results and discussion

### Predicted rice interactions

**Figure 1:**
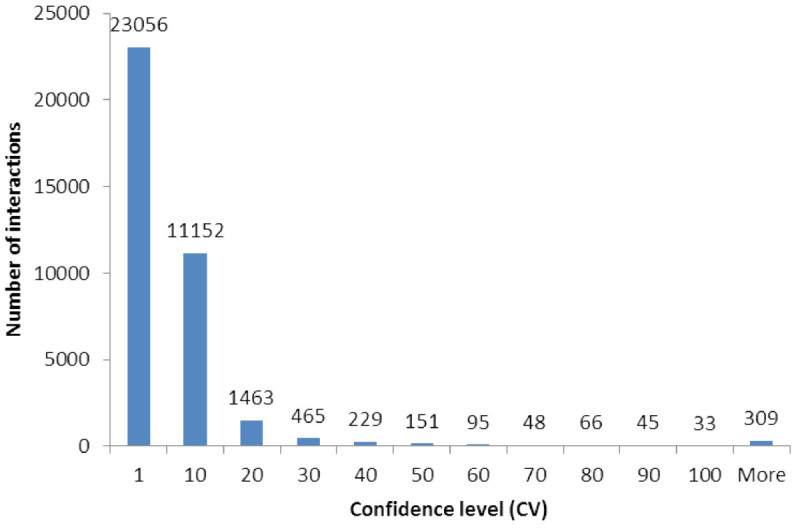
**Confidence Values of predicted rice protein-protein interactions.** Confidence value was calculated from the overall supporting evidence with a multiplier for interactions found by different experimental methodologies and found in different reference species. Most predicted interactions were of low confidence (1), but 11152 interactions had medium and 2904 had high confidence levels. 37112 unique interactions involving 4567 proteins were predicted which included 1671 self interactions and 35441 hetero-interactions.

In this study, a rice protein-protein interaction network was predicted based on the universality of conserved protein function among different organisms. This was undertaken with the assumption that evolutionarily conserved orthologous proteins are likely to retain their interactions with other similarly conserved proteins. Using ortholog prediction algorithm, 13070 rice genes (23% of rice genome) had an ortholog that matched at least one of the eleven reference organisms (*Arabidopsis thaliana*, *Homo sapiens*, *Mus musculus*, *Rattus norvegicus*, *Drosophila melanogaster*, *Caenorhabditis elegans, Saccharomyces cerevisiae*, *Schizosaccharomyces pombe*, *Escherichia coli, Bacillus subtilis, Helicobacter pylori*). A confidence value (CV, see Methods) was calculated to estimate the strength of experimental support for each predicted interaction. We have identified 37112 predicted interactions for 4567 rice proteins (Additional file 1: Table S1), whereby 1671 are predicted self interactions and 35441 are interactions between different proteins. These interactions were classified into 2904 interactions with high confidence (CV > 10), 11152 interactions with medium confidence (CV between 2 and 10) and 23056 interactions with low confidence (CV = 1) (Figure 1). Table 1 lists the twenty protein interactions with the highest confidence, owing to repeatability using different experimental techniques and species. Among these interactions are DNA repair pathways involving RAD50, 51, 54, MCM2, 5, 6, UVH1, MRE11, and others; Cell cycle control pathways with CDC2, cyclin dependent kinases, SNF1-related protein kinase; and the transcription initiation complex. While many of these interactions have been well studied in human and yeast, equivalent studies are lacking for most of these in rice. All genes in Table 1 have been functionally annotated in rice only by sequence homology, often only to the level of general gene family and not their specific role. By adding predicted interactions, the exact role for each protein in the network can be more specifically hypothesized.

**Table 1:** **Twenty rice protein interactions with the highest confidence**

Locus A*	Locus B*	Protein A	Protein B	CV
Os11g40150	Os11g40150	DNA repair protein RAD51 homolog A	DNA repair protein RAD51 homolog A	2080
Os03g53960	Os10g28040	transcriptional adaptor	general control of amino acid synthesis protein 5-like 2	2048
Os01g72880	Os02g37920	MUTL protein homolog 1	ATP binding protein	2040
Os02g30800	Os05g06840	DNA polymerase family B, exonuclease domain containing protein, expressed	DNA polymerase epsilon subunit 2	1404
Os07g02350	Os10g41520	casein kinase II subunit alpha-2	casein kinase II subunit beta-4	1368
Os03g63940	Os05g45420	protein kinase AKINbetagamma-2	SNF1-related protein kinase catalytic alpha subunit KIN10	1281
Os02g29464	Os04g54340	DNA repair protein RAD50	double-strand break repair protein MRE11	1254
Os02g38340	Os08g28190	actin-like protein 3	actin-like protein 2	1155
Os04g43300	Os05g43610	ATBRCA1	ATBRCA1	1040
Os06g43790	Os12g39070	HAF01	TATA-binding protein 2	1026
Os02g52510	Os11g40150	DNA repair protein RAD54-like, putative	DNA repair protein RAD51 homolog A	952
Os03g46650	Os05g26890	guanine nucleotide-binding protein beta subunit	guanine nucleotide-binding protein alpha-1 subunit	936
Os11g29380	Os12g37400	DNA replication licensing factor Mcm2	PROLIFERA protein	800
Os03g01100	Os10g37490	DNA repair endonuclease UVH1	mating-type switching protein swi10	783
Os01g08540	Os05g19270	DNA mismatch repair protein MSH6-2	DNA mismatch repair protein MSH2	774
Os01g32750	Os03g29470	TBP-associated 59 kDa subunit protein	transcription initiation factor TFIID subunit 9B	756
Os01g64820	Os12g13950	DNA polymerase alpha catalytic subunit	DNA polymerase alpha subunit B	720
Os01g07110	Os11g40150	BRCA2 repeat family protein	DNA repair protein RAD51 homolog A	714
Os06g08770	Os07g08170	ruvB-like 2	ruvB-like 1	651
Os03g02680	Os03g05300	cell division control protein 2 homolog 1	cyclin-dependent kinases regulatory subunit	648

*rice locus identifiers are also written as e.g. LOC_Os11g40150.

### Conservation in interactions

The same protein interactions were found in many species, despite the incompleteness of the experimentally-determined interactomes (Wiles et al. 2010). The same pathway interactions for DNA repair is found in 5 other eukaryotic species, including the pathway components RAD51, DNA replication licensing factors MCM2,5,6 and PROLIFERA; DNA polymerase subunits and actin-like proteins (Table 2). These are also interactions with the highest confidence (with CV more than 400). We identified 793 interactions that were present in at least two different species and orthologous genes were found in rice. Proteins in rice with highest connectivity (number of different interactions) were not always the most evolutionarily conserved, which differs from the predicted Arabidopsis interactome (Geisler-Lee et al., 2007). Three of the most conserved protein interactions in rice between DNA polymerase catalytic subunit and DNA polymerase subunit B, small nuclear ribonucleoproteins SmD2 and F and self-interaction of UTP-glucose-1-phosphate uridyltransferase were also among the most conserved in Arabidopsis (Geisler-Lee et al., 2007). The other highly conserved interactions between the two species are different (Table 2). More rice orthologs (13,070) were identified in comparison to Arabidopsis (10,776) possibly owing to a larger genome in rice, and there was an increased pool of reference species.

**Table 2:** **Twenty most conserved rice protein interactions**

Locus A*	Locus B*	Protein A	Protein B	Species	CV
Os11g40150	Os11g40150	DNA repair protein RAD51 homolog A	DNA repair protein RAD51 homolog A	5	2080
Os02g55410	Os11g29380	DNA replication licensing factor MCM5	DNA replication licensing factor Mcm2	5	560
Os01g64820	Os12g13950	DNA polymerase alpha catalytic subunit	DNA polymerase alpha subunit B	5	720
Os02g38340	Os08g28190	actin-like protein 3	actin-like protein 2	5	1155
Os07g22400	Os12g13950	DNA primase large subunit	DNA polymerase alpha subunit B	5	450
Os01g36390	Os11g29380	DNA replication licensing factor mcm4	DNA replication licensing factor Mcm2	5	420
Os11g29380	Os12g37400	DNA replication licensing factor Mcm2	PROLIFERA protein	5	800
Os05g14590	Os11g29380	DNA replication licensing factor MCM6	DNA replication licensing factor Mcm2	5	595
Os01g72880	Os02g37920	MUTL protein homolog 1	ATP binding protein	4	2040
Os09g38030	Os09g38030	UTP--glucose-1-phosphate uridylyltransferase	UTP--glucose-1-phosphate uridylyltransferase	4	176
Os05g24970	Os11g43620	small nuclear ribonucleoprotein Sm D2	small nuclear ribonucleoprotein F	4	272
Os01g71990	Os01g71990	pyrroline-5-carboxylate reductase	pyrroline-5-carboxylate reductase	4	144
Os05g39850	Os11g29380	DNA replication licensing factor MCM3	DNA replication licensing factor Mcm2	4	476
Os02g55410	Os05g39850	DNA replication licensing factor MCM5	DNA replication licensing factor MCM3	4	448
Os07g02350	Os10g41520	casein kinase II subunit alpha-2	casein kinase II subunit beta-4	4	1368
Os03g53960	Os10g28040	transcriptional adaptor	general control of amino acid synthesis protein 5-like 2	4	2048
Os06g43790	Os06g44030	HAF01	transcription initiation factor TFIID subunit 5	4	580
Os02g52510	Os11g40150	DNA repair protein RAD54-like	DNA repair protein RAD51 homolog A	4	952
Os01g53600	Os09g33930	farnesyltransferase beta subunit	farnesyltransferase/geranylgeranyltransferase type IA	4	456
Os05g32310	Os08g08040	SAD1	LSM7 homolog, U6 small nuclear RNA associated	4	224

*rice locus identifiers are also written as e.g. LOC_Os11g40150.

### Experimental verification of predicted rice interactions

A gold standard of 675 experimentally confirmed interactions was abstracted from IntAct (http://www.ebi.ac.uk/intact/; Additional file 2: Table S2). Of these 675 experimentally-determined interactions, there were 168 interactions that were also predicted by our method. This is a very high overlap, with a significance p-value of less than 10^-99^ when compared to a chance overlap with an equal number of random protein pairs. This is especially significant when considering not all experimentally-determined interactions were also evolutionarily conserved, and thus not easily detectable by interacting orthologs. A PubMed ID was assigned to each of the gold standard interactions in our data sets. The remaining experimentally-determined interactions were loaded into the Rice Interactions Viewer (RIV) database.

### Interactome topology

The rice interacting proteins were divided into free ends (981 proteins with single interactions), pipes (499 proteins with two interactions) and hubs of different sizes ranging from three to more than 100 interactions. The largest class of proteins is that of medium hubs (between 11 to 50 interactions). The interacting proteins had an average of 14–15 partners, which is comparable to *Drosophila* (9 interacting partners) and Arabidopsis (11 interacting partners) and smaller than in yeast (22 interacting partners). Table 3 lists the most highly connected rice protein interaction hubs, including heat shock protein 81–1, elongation factor 1-alpha, vacuolar ATP synthase subunit B isoform 1. Among these proteins, ubiquitin-conjugating enzyme E2, ubiquitin-like protein SMT3, fibrallarin-2 were also found among the twenty most highly connected Arabidopsis interaction hubs (Geisler-Lee et al., 2007).

**Table 3:** **Twenty most highly connected rice protein interaction hubs**

Locus	Protein Description	Edges
Os08g39140	heat shock protein 81-1	686
Os03g08050	elongation factor 1-alpha	410
Os06g37180	vacuolar ATP synthase subunit B isoform 1	298
Os01g38970	carbamoyl-phosphate synthase large chain	276
Os07g08330	60 S ribosomal protein L4	272
Os01g62840	mannose-1-phosphate guanyltransferase	245
Os01g73310	actin-1	234
Os01g62244	ubiquitin-conjugating enzyme E2 7	224
Os10g32550	chaperonin CPN60-1, mitochondrial precursor	214
Os11g04070	60 S acidic ribosomal protein P0	197
Os07g31370	ras-related protein Rab-6A	187
Os07g43360	histone acetyltransferase MYST1	182
Os05g38530	heat shock cognate 70 kDa protein	180
Os03g45920	tubulin beta-8 chain	170
Os09g38020	histone H4	169
Os06g38470	histone deacetylase	168
Os11g14220	tubulin alpha-3 chain	167
Os01g68940	ubiquitin-like protein SMT3	167
Os05g08360	fibrillarin-2	162
Os03g13970	26 S proteasome non-ATPase regulatory subunit 4	161

### Protein domain enrichment in rice interologs

Protein-Protein interactions are often mediated by protein interaction domains (PIDs) which bind to other domains (domain-domain interactions), or short conserved amino acid motifs in the partner protein. PIDs that interact most frequently among the rice protein pairs (interologs) were determined using annotation by PFAM domain models (see methods). In rice, the most enriched domains among the interactors included known interaction domains PF00400 (WD40), PF00069 (protein kinase), PF00270 (DEAD), and PF02985 (HEAT). Looking at domain-domain interactions amongst interacting pairs, domain pairs (with one domain in each interacting protein) with extremely high fold of enrichment (vs. random pairing) were often otherwise rare domains among the protein interactions. Among the twenty most frequent PFAM interacting pairs were 8 self interactions between homodomains and 12 interactions between different PFAM domains. Of these 14 (70%) were enriched more than 2 fold, with the highest fold of enrichment (134 fold) for interaction between the homodomains of the proteasome (PF00227; Table 4). The proteasome is a proteinase complex involved in an ATP/ubiquitin-dependent non-lysosomal proteolytic pathway which liberates the cell of misfolded or damaged proteins and controls the level of certain regulatory proteins (Goldberg and Rock, 1992; Hilt and Wolf, 1996; Rivett, 1993; Wilk, 1993). Since its function is crucial and is composed of about 28 distinct subunits that form a highly ordered structure in eukaryotes, it is not surprising that this PFAM domain shows the highest fold of enrichment in the predicted rice interactions. These interactions form a distinct subnetwork of highly conserved interactions.

**Table 4:** **Twenty most frequent domain pairs between interacting partners**

Domain IDs in interacting pairs	Domain names	Number of interactions observed	Fold enrichment vs. random
PF00400 PF00400	WD40 : WD40	166	2.4280
PF00271 PF00270	Helicase C : DEAD	131	8.0228
PF00069 PF00069	Pkinase : Pkinase	120	0.0383
PF00227 PF00227	Proteasome : Proteasome	114	133.8151
PF00076 PF00076	rrm :rrm	112	1.2412
PF01486 PF00319	K-box : SRF-TF	104	30.0497
PF00271 PF00271	Helicase_C : Helicase_C	101	3.5858
PF00400 PF00271	WD40 : Helicase_C	99	2.2560
PF00271 PF00076	Helicase C: rrm	94	1.8646
PF01423 PF01423	Sm : Sm	91	116.3077
PF07714 PF00069	Pkinase_Tyr : Pkinase	91	0.0473
PF00270 PF00076	DEAD : rrm	74	2.5320
PF00400 PF00069	WD40 : Pkinase	70	0.1512
PF00400 PF00076	WD40 : rrm	70	0.8912
PF00069 PF00036	Pkinase : efhand	70	0.1994
PF02985 PF00400	HEAT : WD40	65	2.5236
PF00400 PF00118	WD40 : cpn60_TCP1	62	8.8624
PF00004 PF00004	AAA : AAA	60	3.4780
PF00400 PF00270	WD40 : DEAD	59	2.3192
PF00270 PF00270	DEAD : DEAD	58	6.1273

### Predicted rice interactome subnetworks

**Figure 2:**
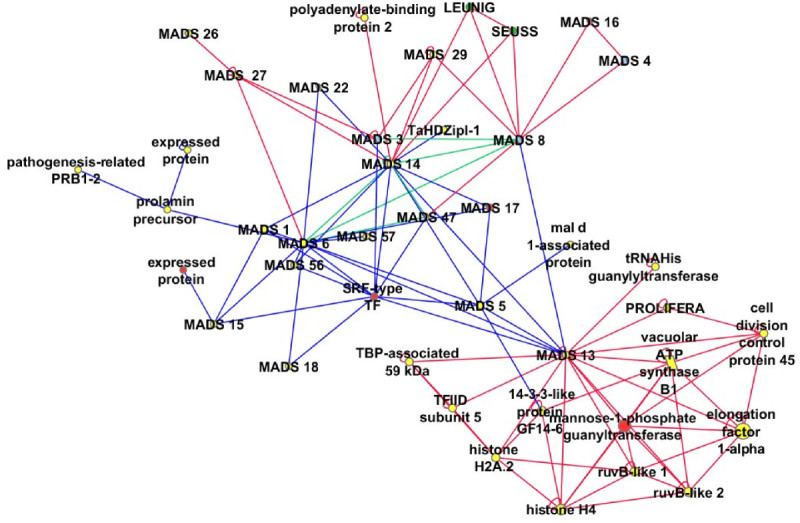
**MADS network in rice expanded by predicted interactions .** The core interactions between rice MADS box proteins were experimentally determined by de Folter et al. 2005(Circles: rice proteins, Blue edges: experimentally determined interactions). This network is expanded by predicted interactions (Red edges), and several experimentally determined interactions were also predicted (Green edges).

**Figure 3:**
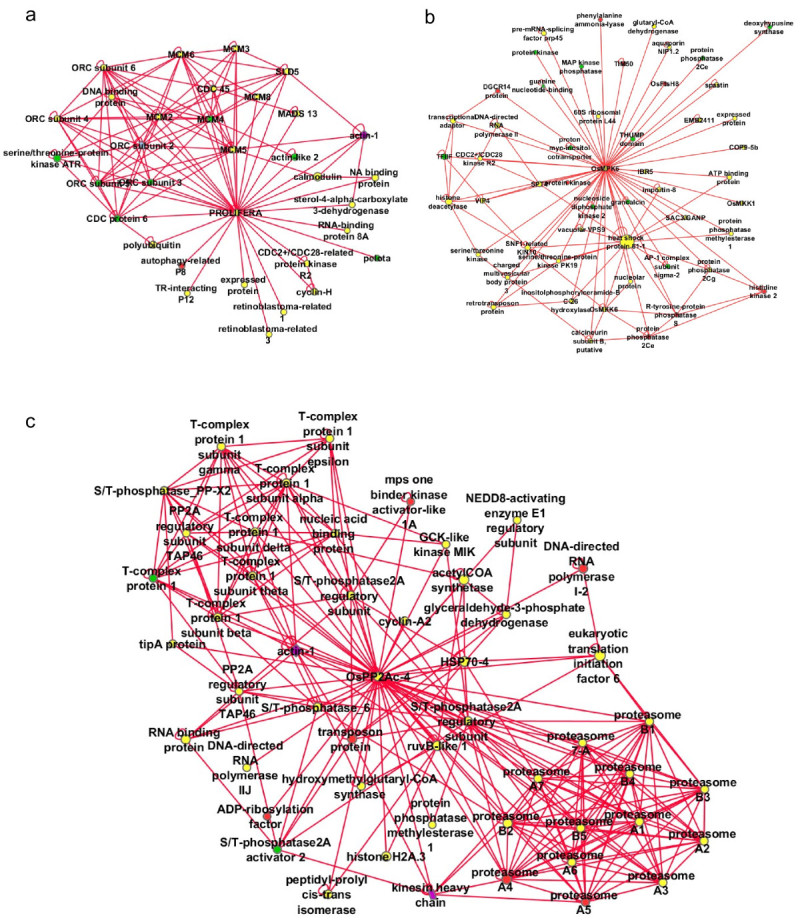
**Subnetworks of predicted rice interactions.** Subnetworks were constructed by identifying protein that interact with a single protein (**a**: PROLIFERA), or interactions involving proteins with a defined domain and their interacting neighbors (**b**: MAP kinase, **c**: protein phosphatase 2 C isoform 4).

In this study, we present a predicted interactome from rice that is useful for hypothesis generation towards better understanding of protein-protein interactions in rice and also other monocotyledonous plants. We have constructed a predicted rice MADS network consisting of 19 rice MADS-box family members which was determined partially by experiments in rice (Figure 2) which has been expanded using interologs from Arabidopsis, (de Folter et al. 2005) indicated by blue lines in Figure 2. Although these genes are plant specific, the degree of interconnectivity among MADS box genes is not surprising as many proteins form functional complexes consisting of homodimers or heterodimers (Pelaz et al., 2000; Theissen and Melzer, 2007) that have diverse roles. In addition, predicted interactions have connected several rice MADS proteins to proteins that do not belong to the MADS-box family, e.g. LEUNIG (LUG), SEUSS (SEU), and PROLIFERA and more than 10 other proteins involved in diverse functions including DNA metabolism (RuvB-like proteins; Gorynia et al., 2006), initiation of transcription TATA binding protein protein associated factors (TAFs) and a general transcription factor, TFIID (Cler et al., 2009), cell cycle progression (mannose-1-phosphate guanyltransferase, Donoso et al., 2005), and cell division (cell division control protein 45). Both LUG and SEU are known transcription co-repressors that form a co-repressor complex with MADS box dimers, APETALA1 (AP1) AP1- AGAMOUS-LIKE 24 (AGL24) and AP1-SHORT VEGETATIVE PHASE (SVP) to repress *AGAMOUS* (*AG*) gene in flowers (Gregis et al., 2006). SEU has been demonstrated to interact with AP1 and SEPALLATA3 (SEP3) to bridge the interaction between AP1/SEP3 and LUG in *Arabidopsis* (Sridhar et al., 2006) resulting in transcription repression during flower development. OsMADS14 and OsMADS8 which are connected to SEU here, could be the rice orthologs for AP1 and SEP, while OsMADS13 could be a rice ortholog for AGL11 (Arora et al., 2007) which was reported to be preferentially expressed in ovule (Rounsley et al. 1995) and carpel (Yung et al., 1999). OsMADS13 was also connected to PROLIFERA (PRL) which encodes a DNA replication licensing factor Mcm7. In Arabidopsis, *PRL* was demonstrated to be expressed in dividing cells in the palisade layer of the leaf, founder cells of initiating flower primordial, and central cell nucleus of mature mega gametophytes (Springer et al., 2000) whereas PROLIFERA protein was reported during G(1) phase of the cell cycle. The interaction network involving PROLIFERA was further expanded through interologs in other eukaryotes (Figure 3a), showing the relationship between the MADS box network to conserved network of cell division and cell cycle regulators.

The predicted rice interactome is also useful for the functional annotation of expressed proteins with unknown functions through their associations with known proteins, or known pathways in the predicted network. Subnetworks of interacting proteins were constructed around MAP kinase signaling proteins identified by homology (Figure 3b) and the known protein phosphatase OSPP2C4, (Figure 3c). These subnetworks contained many expected interactions but also included both metabolic enzymes (e.g. acyl co-enzyme A synthase in Figure 3c) and regulators (e.g. serine/threonine phosphatase 2A regulatory subunits), as well as unexpected connections between metabolic enzymes, signaling and ribosomal proteins.

### Coexpression of interologs

**Figure 4:**
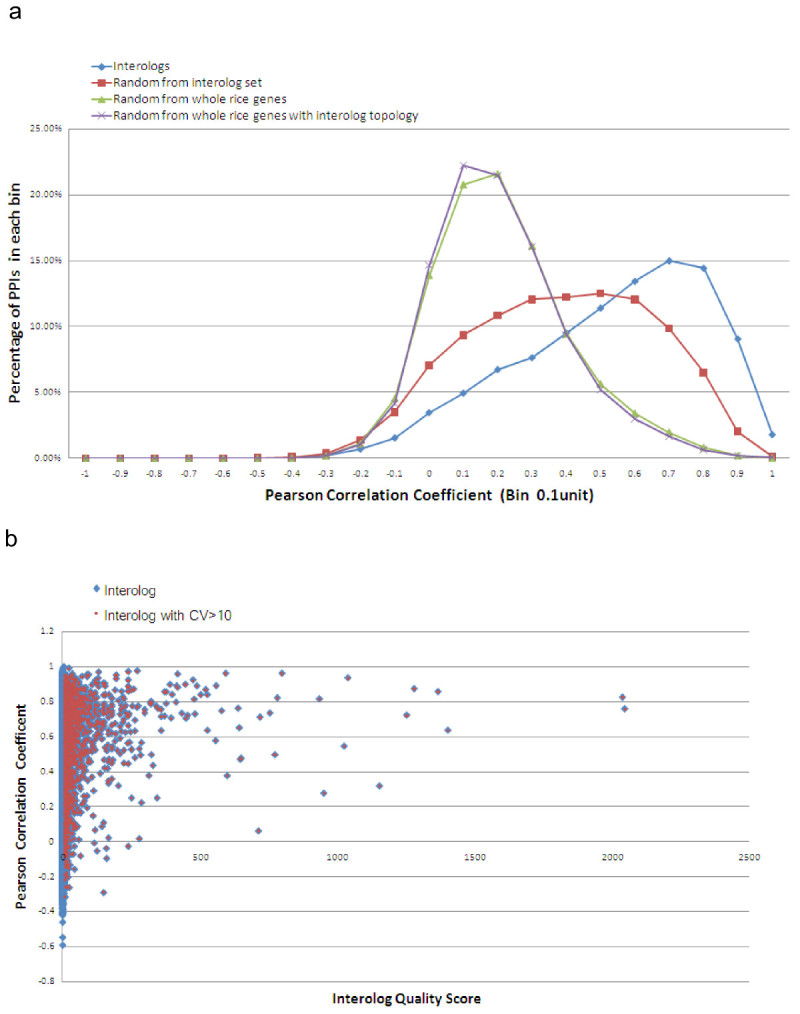
**Co-expression of interologs. a**, The PCC for 37,112 predicted interaction pairs was calculated and plotted as the number of pairs in each Pearson correlation coefficient range (blue curve). The PCC calculation is also performed for 37,112 randomly selected pairs of rice genes from within our interactome (red curve), from the whole rice genes (green curve), or from whole rice genes such that the topology of the random network was the as that of our predicted interactome in terms of node degree distribution (purple curve). **b**, The interolog CV was plotted against the PCC for each pair (blue dots). The red dots are the interologs whose CV is larger than 10.

Proteins which physically interact are often expressed at the same time in the same tissue (Narayanan et al. 2010). This is possibly due to common transcription factors controlling individual members of a protein complex or pathway, a common network motif known as a single input module (Alon, 2007). Especially in metabolism, co-expressed enzymes utilize “just in time” mechanisms of regulation so that they do not waste energy producing idle proteins (Zaslaver et al., 2004). Thus mRNA levels of the transcripts for interacting proteins also frequently co-vary, as do levels of transcripts for proteins that are part of a signaling chain or enzymes in a metabolic pathway that is coordinately stimulated or suppressed. A network of gene co-expression can itself be informative in pathway reconstruction. As a result, if a pair of proteins is positively coexpressed, the confidence level to predict the pair interacts is generally high (Geisler-Lee et al. 2007). The co-expression level of each interolog is measured by Pearson Correlation Coefficient (PCC) which is computed from available rice Affymetrix microarray data which includes 165 data sets in total from diverse tissues and treatments (Additional file 3: Table S3). In comparison to the randomly generated gene pairs (see methods), our predicted interologs exhibited a strong and statistically significant trend of coexpression (Figure 4). Predicted interologs with low coexpression were also found in the analysis. Not all proteins with physical interactions are co-expressed, as may be the case with rate limiting steps or proteins regulated at the post-transcriptional level. One protein can be constitutively expressed while the other interacting partner is expressed under certain conditions only, especially where a pathway branches into two downstream paths. Protein relocation to another compartment or even another tissue is also a possible cause. Collectively, these are called “limiting factor” based regulation mechanisms. Thus lack of expression correlation does not necessarily imply that proteins do not interact. Indeed the combination of co-expression and predicted physical interaction reveals the regulation mechanisms involved in that pathway as either “just in time”, or “limiting factor” based. Overall, however, a positive correlation was found between the interolog coexpression (PCC) and the interolog confidence level (CV), indicating that “just in time” regulation is more common.

### Subcellular localization of interologs

**Figure 5:**
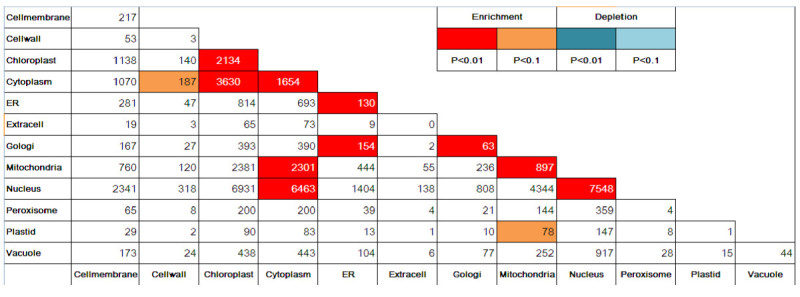
**Co-localization of interologs.** Analysis of all interaction protein pairs in which both partners were assigned to a subcellular compartment. Compartment pairs that showed enriched or depleted numbers of interactions (compared to chance) are color coded.

In a general sense, for a pair of proteins to physically interact, each protein must be located at same or adjacent subcellular compartments either permanently or transiently. Thus it is foreseeable that interacting proteins are more likely to have the same or adjacent subcellular localization. We have performed subcellular localization enrichment analysis to validate this trend. Unlike the similar approach for Arabidopsis where extensive subcellular localization information can be retrieved from SUBA (Geisler-Lee et al. 2007), comprehensive experimentally derived subcellular localization data for rice are not available. Fortunately, many computational methods are available for predicting protein subcellular localization. Some machine learning-based methods that use sequence-derived features to predict localization have reached remarkable levels of performance. Therefore, we adopted one of these computational methods named Plant-mPloc (http://www.csbio.sjtu.edu.cn/bioinf/plant-multi/; Chou and Shen, 2010) to predict the subcellular localization of each protein in our interactome. Since Plant-mPloc is able to cover 12 distinct compartments, 78 distinct compartment pairs were used for analysis. Figure 5 shows the observed number of interologs of each possible subcellular compartment pairs. P values associated with the deviation of these numbers from a random interactome network with the same properties (see “Material and Methods”) were used to evaluate the statistical significance of enrichment or depletion. Most of the statistically significant enriched compartment pairs are those paired with same compartment or adjacent compartments such as ER and Golgi (Figure 5). A significant number of cytosolic localized proteins are predicted to interact with nuclear and mitochondrial localized proteins, indicating possible import/export regulation and dual localized proteins such as transcription factors. No statistically significant depletion was found in this analysis. The results differ slightly from what we observed in the case of the predicted interactome for Arabidopsis (Geisler-Lee et al., 2007), in part due to the large number of proteins in our interactome that have multiple predicted subcellular localizations while in the Arabidopsis approach single subcellular localization was assigned to each protein through winner-takes-all strategy. We were unable to use the same strategy since Plant-mPloc is our only source for subcellular localization determination. In our analysis, one predicted interaction is scored as belonging to several compartment pairs thereby increasing the number of interologs in these compartment pairs, which could cause no depletion. Additionally, the accuracy of the Plant-mPloc prediction must also be considered. Nevertheless, a clear tendency that interologs are more likely enriched in the compartment pairs paired with same or adjacent compartment can be found from our analysis.

### Rice interactome visualization

**Figure 6:**
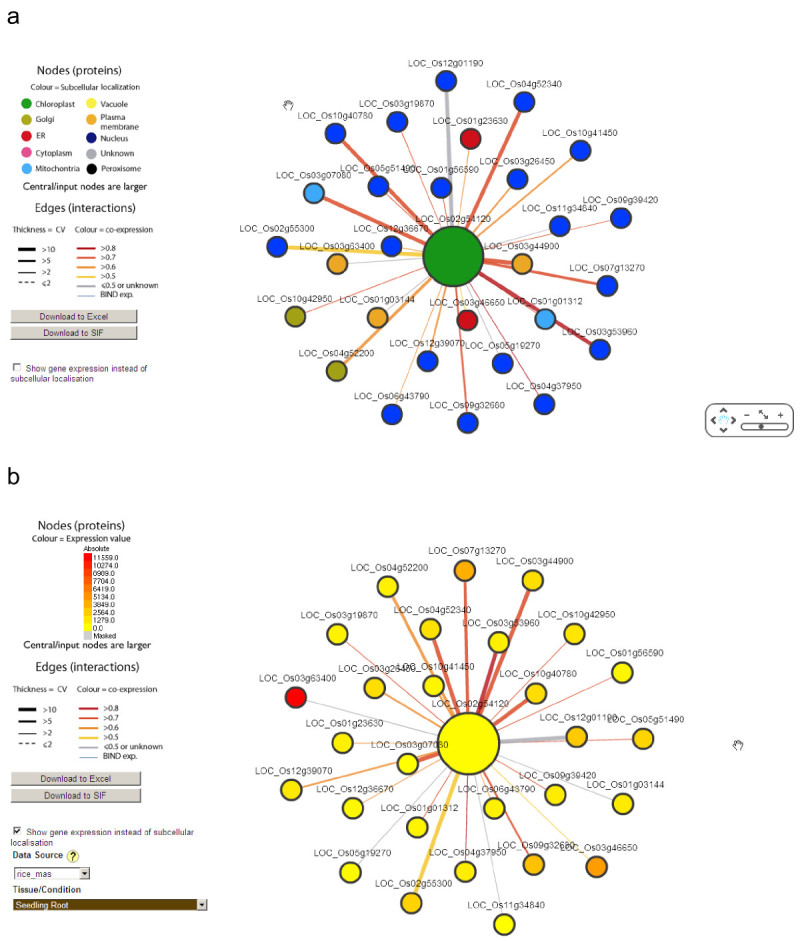
**Visualizing the rice interactome by RIV. a**, nodes are colored according to its subcellular localization, edges are colored according to its coexpression correlation and the line thickness correlates to CV. The popup message is the ID, annotation and subcellular localization of the clicked node. **b**, same as a except the nodes color represent the expression level in “Seeding Root” tissue.

A predicted interactome is usually stored in a table with columns and rows. However, in essence it is a network which consists of nodes and edges, which is better visualized as a graph. The Rice Interactions Viewer (RIV; Figure 6), which was developed based on the infrastructure of Arabidopsis Interactions Viewer (AIV), is such a web–based interactome network visualization tool that allows users to perform customized query and analysis (Geisler-Lee et al. 2007). Besides the replacement of the AIV data set with the rice interolog data set described here and the amendment of GUI text, some redundant code was also corrected for the purpose of functional expansion. One major improvement on RIV is the integration of CytoscapeWeb (Lopes et al., 2010; http://cytoscapeweb.cytoscape.org/). By using the graph definition language GraphML, the color of each protein (node) is painted differently based on its predicted subcellular localization (Figure 6a). Alternatively, nodes can be colored according to its expression level in a specific tissue/condition (Figure 6b), based on rice gene expression data sets stored in the Bio-Array Resource for Plant Biology (Toufighi et al., 2005). For each edge which connects a pair of proteins, the RIV is able to show different color based on the coexpression value (PCC). As well the relative thickness corresponds to its Confidence Value (CV). Moreover, a simple mouse click on each protein (node) is able to show its ID, annotation and predicted subcellular localization.

## Conclusions

### How to use the predicted interactome

A rice interactome has been predicted based on conservation of protein interactions across species over the course of evolution. Each interaction has been assigned a confidence level (CV) as an internal quality control. Assignments for subcellular localization and coexpression can be used to further validate the predicted interaction. Coexpression analysis has shown that interacting proteins tend to have similar expression profiles, and tend to be localized to the same or adjacent compartment. By inputting rice proteins of interest, the Rice Interactions Viewer (Figure 6) can be used to display the results of a query of our predicted interactome in a tablular format and CytoscapeWeb network graph format. In table format output, for the interologs confirmed by published literature, users can link to the paper by clicking the assigned PubMedID. Users can also sort any column easily by clicking the column header. This function is very useful for users who want to check the highest coexpression or confidence level in the output of interologs. This predicted interactome is not without limits however, and some caution should be observed before concluding on any positive and especially negative results (interactions expected but not found). Firstly, the interactions predicted are based on orthology between rice and other eukaroytes with experimentally-determined interactomes. Other than about 4000 interactions in Arabidopsis, no other plant has a large number of experimentally-determined interactions to draw from. With the possible exception of yeast (*S. cerevisiae*), none of the experimental data sets from reference organisms are complete. The interactions contained in this data set are of evolutionarily conserved proteins and pathways. However as the experimental data are based on high throughput studies, the predicted rice interactome does not represent only well studied interactions like the proteosome, ribosome and base transcriptional machinery: there are still some surprising connections and a number of unknown proteins within the data set as well.

Note added in revision: we expanded the number of predictions by incorporating Braun et al.’s Arabidopsis Interactome data set (Arabidopsis Interactome Mapping 2011) and also from an Arabidopsis G-protein network (Klopffleisch et al. 2011). A predicted rice interactome by Gu et al. (2011) was published while this manuscript was under review. While Gu et al. used similar methodologies, our study had significantly more reference species and thus has improved resolution of conserved interactions.

## Methods

### Prediction of orthologs

Peptide sequences from rice (*Oryza sativa* subsp. japonica), *Arabidopsis thaliana*, *Homo sapiens*, *Mus musculus*, *Rattus norvegicus*, *Drosophila melanogaster*, *Caenorhabditis elegans, Saccharomyces cerevisiae*, *Schizosaccharomyces pombe*, *Escherichia coli, Bacillus subtilis, Helicobacter pylori* were retrieved from TAIR (http://www.arabidopsis.org; Swarbreck et al., 2008), ENSEMBL (http://www.ensembl.org/index.html; Flicek et al., 2010) for the prediction of orthologs using INPARANOID 3.0 (inparanoid.cgb.ki.se, O'Brien et al. 2005) at BLOSUM 80 for rice-*A. thaliana*, BLOSUM 80 and 62 for rice- *H. sapiens*, *M. musculus*, *R. norvegicus* and *S. cerevisiae*; BLOSUM 62 for *D. melanogaster*, *C. elegans, Sch. pombe* and BLOSUM 45 for *E. coli, B. subtilis* and *H. pylori.* Only ortholog pairs that had a score of 100% confidence (From INPARANOID 3.0) were retrieved for the prediction of rice interactome.

### Mapping of rice orthologs to reference interactomes

Interactome databases were obtained from BioGRID (version 47; http://www.thebiogrid.org; Breitkreutz et al., 2008), DIP (October 2008 release; dip.doe.mbi.ucla.edu; Salwinski et al., 2004); and IntAct (downloaded December 5, 2009, http://www.ebi.ac.uk/intact; Aranda et al., 2010), respectively. Rice orthologs were mapped onto interactome data using a cross-reference tables assembled from identifiers retrieved from ENSEMBL, The *Arabidopsis* Information Resource (TAIR; Swarbreck et al., 2008) and The Institute for Genomic Research (JCVI; http://www.jcvi.org; formerly TIGR). A predicted interactome was recorded in an Excel spreadsheet when both interacting proteins in a reference species had orthologs in rice. Duplicates of the same interactions were removed from different interactome data sets and from different reference species to generate unique interacting protein pairs.

### Calculation of the confidence value (CV) for experimental support

The confidence value (CV) for experimental support of a predicted interaction was calculated using the following formula, CV = N x E x S; where N is the total number of data sets in which the interaction was recorded, E is the number of different experimental methods from which an interaction was inferred, and S is the number of reference species from which the same interaction was recorded. The higher the CV, the more likely an interaction is to be conserved in multiple species and to be more convincing as it is supported by different experimental methods. The interactions were classified according to their CV to three groups: low confidence (CV = 1), medium confidence (CV = 2 to 10) and high confidence (CV > 10).

### Mapping of rice orthologs to Gene Ontology (GO)

Gene Ontology (GO) terms for rice were retrieved from the GO site (Revision 1.52 validated 30 August 2008; http://geneontology.org). The top 7 enriched GOSlim terms for molecular functions (MF) and cellular component (CC), respectively; were used as node features in the visualization of protein network using Cytoscape 2.6 (Lopes et al., 2010; chianti.ucsd.edu).

### Mapping of rice orthologs to PFAM domains

The PFAM data of rice proteins were retrieved from Rice Genome Annotation Project from Michigan State University, USA (rice.plantbiology.msu.edu/). Some of the proteins involved in the interactome may possess more than one PFAM domain, thus all possible pairs of PFAM domains of the interacting protein pairs were queried using MySql (Additional file 4: Table S4). The fold enrichment (F) was calculated as the observed number of PFAM pairs in the rice predicted interactions (O) divided by the expected number of PFAM pairs in the rice predicted interactions (E), where E is the total number of PFAM pairs in the rice predicted interactions x (frequency of PFAM domain of protein A x frequency of PFAM domain of protein B found in the total number of unique interacting proteins).

### Subcellular localization prediction and enrichment analysis

The subcellular localization of 67,393 rice proteins was predicted by Plant-mPLoc which covers 12 distinct cellular compartments: chloroplast, nucleus, cytoplasm, mitochondrion, cell wall, cell membrane, peroxisome, endoplasm reticulum, golgi apparatus, vacuole, plastid and extracellular. Due to the existence of splice variants and the nature of protein sorting and trafficking, 8,970 proteins have multiple predicted subcellular localizations. To assess the statistical significance of the enrichment of different subcellular compartment pairs in the predicted rice interactome, we used a randomization algorithm. The P value for the observed numbers of interologs is calculated using a Poisson

1 $$P(n_{\mathrm{ab}})=\{\begin{array}{cc} \sum_{j=0}^{n_{\mathrm{ab}}}n_{\mathrm{ab}}j\frac{e(-\bar{nab})}{j!} & n_{\mathrm{ab}}<\bar{n_{\mathrm{ab}}}(deplation)\\ \sum_{j=n_{\mathrm{ab}}}^{\infty }n_{\mathrm{ab}}j\frac{e(-\bar{nab})}{j!} & n_{\mathrm{ab}}<\bar{n_{\mathrm{ab}}}(enrichmemt) \end{array}$$

distribution:

Here *n*_*ab*_ is the observed number of interologs in our predict interactome where one protein is in compartment ***a*** and the other in ***b***. *n*_*ab*_ is given by:

2 $$n_{\mathrm{ab}}=\sum_{j}\sum_{i<j}(C_{\mathrm{ia}}C_{\mathrm{jb}}OR\;C_{\mathrm{ib}}C_{\mathrm{ja}})eij$$

- *C*_*ia*_ = 1 or 0, whether protein ***i*** is in compartment ***a*** or not
- *C*_*ib*_ = 1 or 0, whether protein ***i*** is in compartment ***b*** or not
- *e*_*ij*_ = 1 or 0, whether protein ***i*** and protein ***j*** interacts or not

Here *n*_*ab*_ is the expected number of interologs with one protein in location ***a*** and the other in location ***b*** for the ensemble of random protein networks that maintain the following properties as the predicted network: the annotation of proteins in their subcellular localization, the degree (*kk*) of each protein (the number of proteins that interact with it), and the total number of interacting pairs (*EE*), *n*_*ab*_ is given by

3 $$\begin{array}{l} \bar{n_{\mathrm{ab}}}=\sum_{j}\sum_{i<j}(C_{\mathrm{ia}}C_{\mathrm{jb}}\;OR\;C_{\mathrm{ib}}C_{\mathrm{ja}})\bar{e_{\mathrm{ij}}}\\ \bar{e_{\mathrm{ij}}}=\frac{k_{i}k_{j}}{\left(2E+k_{1}k_{j}\right)}\;and\;E=\frac{1}{2}\sum_{i}k_{i} \end{array}$$

- *C*_*ia*_ = 1 or 0, whether protein ***i*** is in compartment ***a*** or not
- *C*_*ib*_ = 1 or 0, whether protein ***i*** is in compartment ***b*** or not
- *k*_*i*_*,k*_*j*_ = the node degree of protein(node) *i,j*
- *e*_ij_ = the probability of protein(node) i and protein(node) j interacts
- ***E*** = total number of interactions(edges)

The P values are finally subject to a multiple-test correction ***P***(multi) = 1 − (1−***P***)^m^ where for enrichment ***m*** equals the number of *ab* pairs with at least one observed interolog and for depletion ***m*** equals the number of *ab* pairs possible in the ensemble of random networks.

### Coexpression analysis

Coexpression between interacting proteins was determined using the Pearson correlation coefficient

4 $$r=\frac{1}{N}\sum_{i=1.N}\left(\frac{X_{i}-\bar{X}}{\sigma_{x}}\right)\left(\frac{Y_{i}-\bar{Y}}{\sigma_{y}}\right)$$

Where N is the number of expression samples, X is the expression level for gene X in *i*^th^ sample, and Y is the expression level for gene Y in the *i*^th^ sample (−1≤ ***r*** ≤1).

High positive ***r*** values indicate a correlation of expression patterns, while low negative ***r*** values indicated anti-correlation. A large multi data set compendium consisting of 165 diverse data sets (see Additional file 3: Table S3) was used to generate an accurate representation of gene expression. To determine whether the interolog coexpression distribution was enriched in pairs that exhibited high correlation coefficients, we performed a two-sample Kolomogorov-Smirnov test on the interolog and random distributions. Random distributions were generated by randomly generating protein pairs within the protein collection of our predicted interactome, from any of proteins in whole rice genome, or from any protein in whole rice genome such that the topology of this random set matched that of our predicted interactome in terms of degree distribution.

## Electronic supplementary material

Additional file 1:**Table S1.** The rice predicted interactome. (XLSX 7 MB)

Additional file 2:**Table S2.** PFAM domains for interacting pairs. (XLSX 3 MB)

Additional file 3:**Table S3.** List of GSM identifiers of microarray expression data. (XLSX 17 KB)

Additional file 4:**Table S4.** Experimental verification of predicted interactions. (XLSX 43 KB)

Below are the links to the authors’ original submitted files for images.

Authors’ original file for figure 1

Authors’ original file for figure 2

Authors’ original file for figure 3

Authors’ original file for figure 4

Authors’ original file for figure 5

Authors’ original file for figure 6
