# Supplementary figures and images for: A predicted protein interactome for rice
Source: Rice (N Y). 2012 Jul 2;5:15. doi: 10.1186/1939-8433-5-15 (PMC4883691; doi:10.1186/1939-8433-5-15)

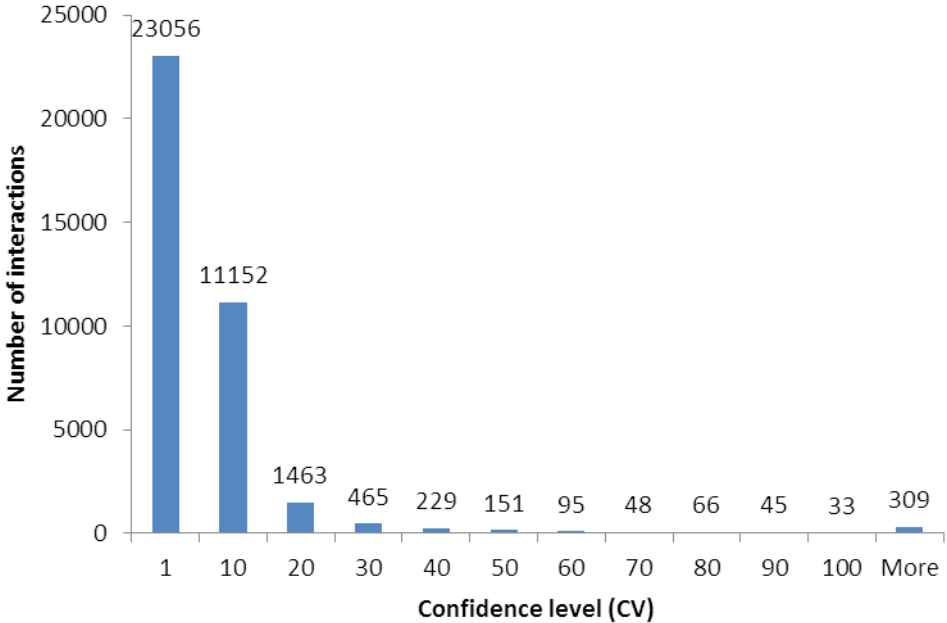

Supplement: Supplementary file 5 — Authors’ original file for figure 1 [file 12284_2012_30_MOESM5_ESM.pdf]

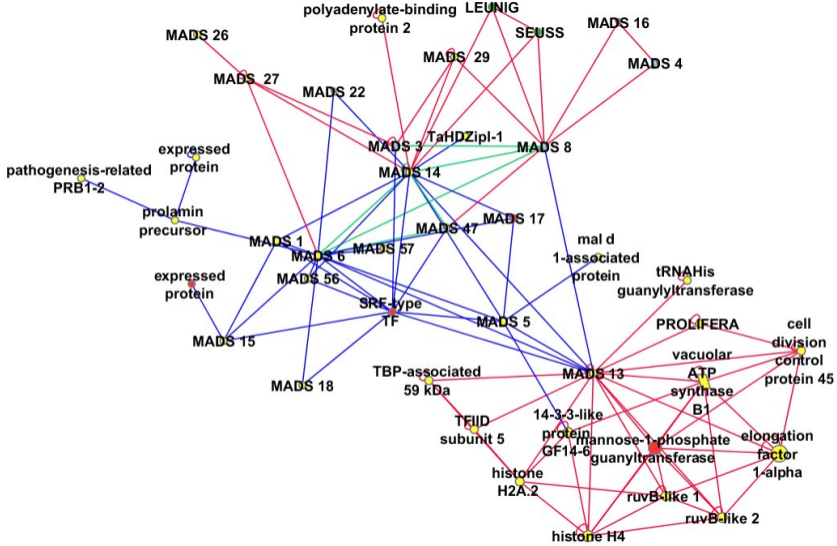

Supplement: Supplementary file 6 — Authors’ original file for figure 2 [file 12284_2012_30_MOESM6_ESM.pdf]

C

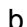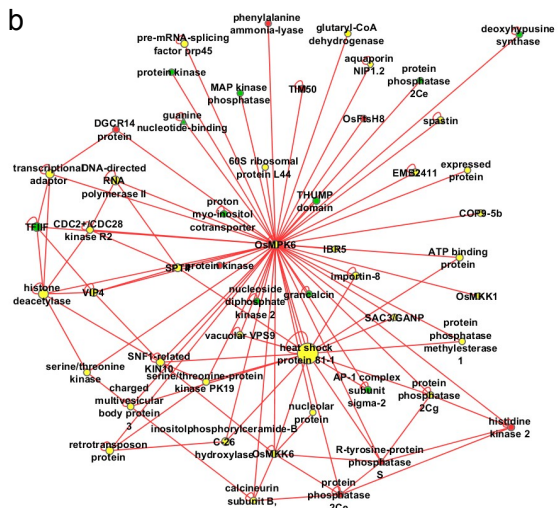

Supplement: Supplementary file 7 — Authors’ original file for figure 3 [file 12284_2012_30_MOESM7_ESM.pdf]

a

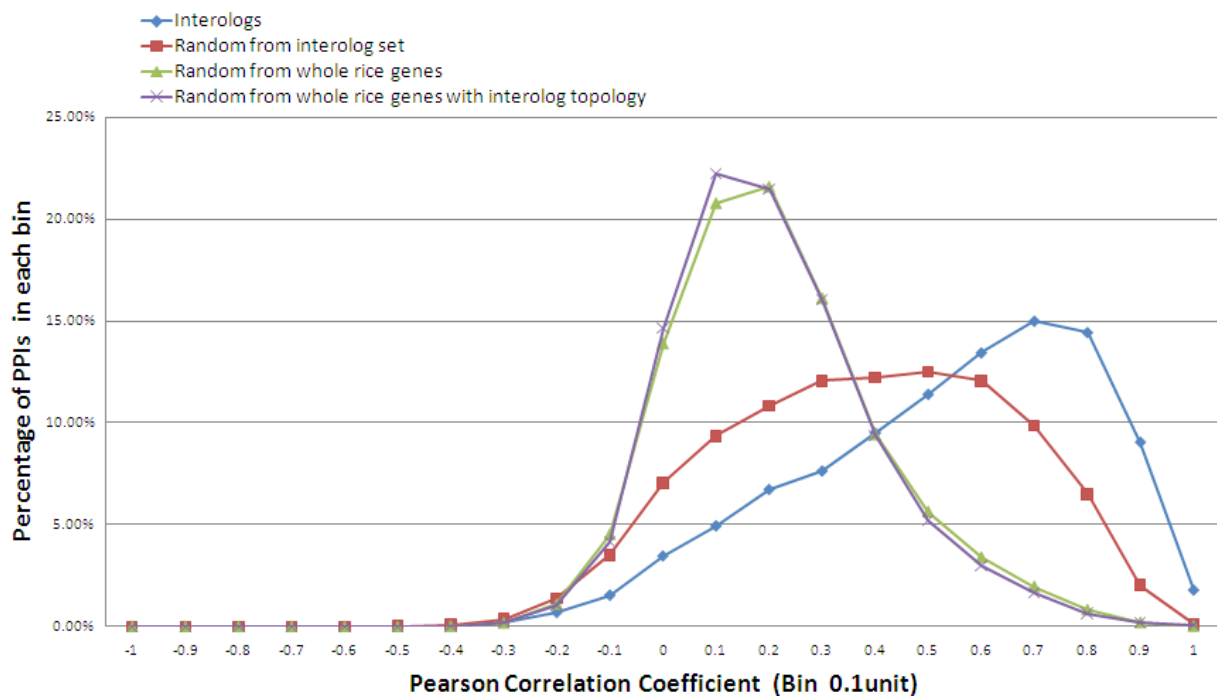

b

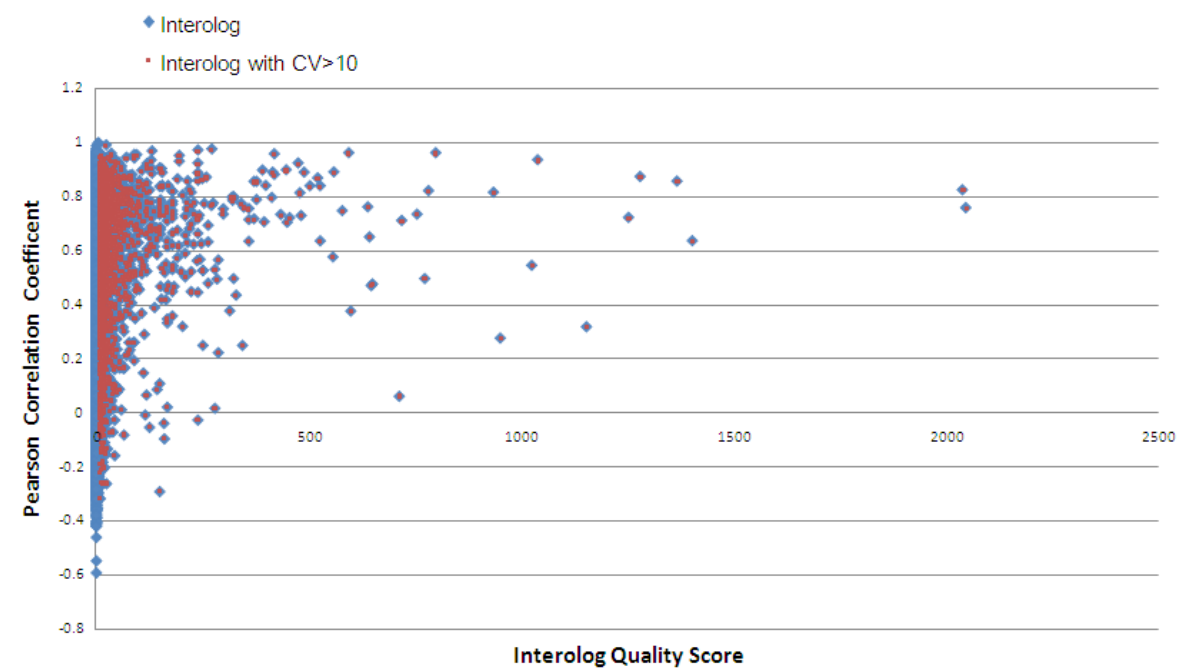

Supplement: Supplementary file 8 — Authors’ original file for figure 4 [file 12284_2012_30_MOESM8_ESM.pdf]

a

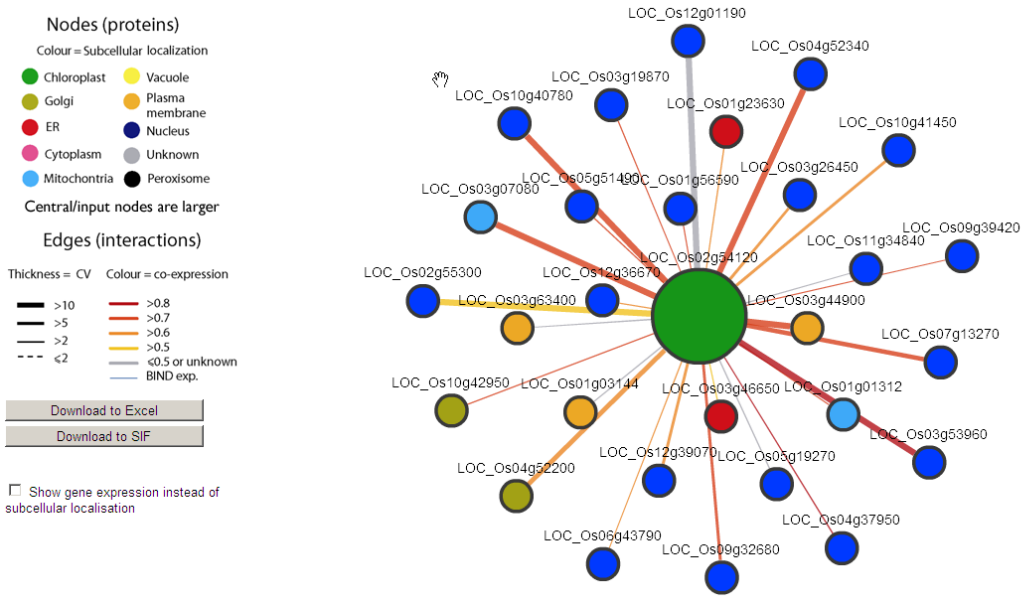

b

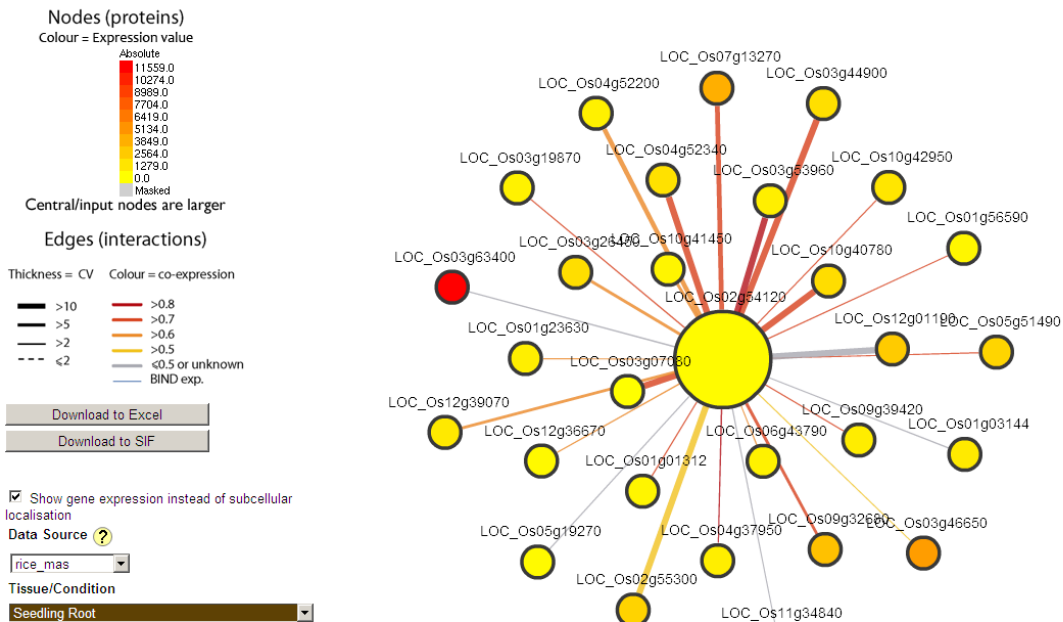

Supplement: Supplementary file 10 — Authors’ original file for figure 6 [file 12284_2012_30_MOESM10_ESM.pdf]
