# Supplementary material for: A predicted protein interactome for rice
Source: Rice (N Y). 2012 Jul 2;5:15. doi: 10.1186/1939-8433-5-15 (PMC4883691; doi:10.1186/1939-8433-5-15)
Supplement: Supplementary file 9 — Authors’ original file for figure 5 [file 12284_2012_30_MOESM9_ESM.pdf]

	Enrichment		Depletion	
	P<0.01	P<0.1	P<0.01	P<0.1

Cellmembrane	217												
Cellwall	53	3											
Chloroplast	1138	140	2134										
Cytoplasm	1070	187	3630	1654									
ER	281	47	814	693	130								
Extracell	19	3	65	73	9	0							
Gologi	167	27	393	390	154	2	63						
Mitochondria	760	120	2381	2301	444	55	236	897					
Nucleus	2341	318	6931	6463	1404	138	808	4344	7548				
Peroxisome	65	8	200	200	39	4	21	144	359	4			
Plastid	29	2	90	83	13	1	10	78	147	8	1		
Vacuole	173	24	438	443	104	6	77	252	917	28	15	44	
	Cellmembrane	Cellwall	Chloroplast	Cytoplasm	ER	Extracell	Gologi	Mitochondria	Nucleus	Peroxisome	Plastid	Vacuole	
